# Enhanced Working Memory Binding by Direct Electrical Stimulation of the Parietal Cortex

**DOI:** 10.3389/fnagi.2017.00178

**Published:** 2017-06-08

**Authors:** Agustina Birba, Eugenia Hesse, Lucas Sedeño, Ezequiel P. Mikulan, María del C. García, Juan Ávalos, Federico Adolfi, Agustina Legaz, Tristán A. Bekinschtein, Máximo Zimerman, Mario Parra, Adolfo M. García, Agustín Ibáñez

**Affiliations:** ^1^Laboratory of Experimental Psychology and Neuroscience (LPEN), Institute of Cognitive and Translational Neuroscience (INCyT), INECO Foundation, Favaloro UniversityBuenos Aires, Argentina; ^2^National Scientific and Technical Research Council (CONICET)Buenos Aires, Argentina; ^3^Instituto de Ingeniería Biomédica, Facultad de Ingeniería, Universidad de Buenos AiresBuenos Aires, Argentina; ^4^Hospital Italiano de Buenos AiresBuenos Aires, Argentina; ^5^Consciousness and Cognition Laboratory, Department of Psychology, University of CambridgeCambridge, United Kingdom; ^6^Department of Psychology, School of Social Sciences, Heriot-Watt UniversityEdinburgh, United Kingdom; ^7^Human Cognitive Neuroscience, Centre for Cognitive Ageing and Cognitive Epidemiology, Alzheimer Scotland Dementia Research Centre, Department of Psychology, University of EdinburghEdinburgh, United Kingdom; ^8^Neuroprogressive and Dementia Network, NHS Research ScotlandEdinburgh, United Kingdom; ^9^Facultad de Psicología, Universidad Autónoma del CaribeBarranquilla, Colombia; ^10^Faculty of Education, National University of Cuyo (UNCuyo)Mendoza, Argentina; ^11^Center for Social and Cognitive Neuroscience (CSCN), School of Psychology, Universidad Adolfo IbañezSantiago, Chile; ^12^Centre of Excellence in Cognition and its Disorders, Australian Research Council (ARC)Sydney, NSW, Australia

**Keywords:** working memory binding, Alzhimer’s disease, direct electrical stimulation, short term memory, single case study

## Abstract

Recent works evince the critical role of visual short-term memory (STM) binding deficits as a clinical and preclinical marker of Alzheimer’s disease (AD). These studies suggest a potential role of posterior brain regions in both the neurocognitive deficits of Alzheimer’s patients and STM binding in general. Thereupon, we surmised that stimulation of the posterior parietal cortex (PPC) might be a successful approach to tackle working memory deficits in this condition, especially at early stages. To date, no causal evidence exists of the role of the parietal cortex in STM binding. A unique approach to assess this issue is afforded by single-subject direct intracranial electrical stimulation of specific brain regions during a relevant cognitive task. Electrical stimulation has been used both for clinical purposes and to causally probe brain mechanisms. Previous evidence of electrical currents spreading through white matter along well defined functional circuits indicates that visual working memory mechanisms are subserved by a specific widely distributed network. Here, we stimulated the parietal cortex of a subject with intracranial electrodes as he performed the visual STM task. We compared the ensuing results to those from a non-stimulated condition and to the performance of a matched control group. In brief, direct stimulation of the parietal cortex induced a selective improvement in STM. These results, together with previous studies, provide very preliminary but promising ground to examine behavioral changes upon parietal stimulation in AD. We discuss our results regarding: (a) the usefulness of the task to target prodromal stages of AD; (b) the role of a posterior network in STM binding and in AD; and (c) the potential opportunity to improve STM binding through brain stimulation.

## Working Memory Binding: Antecedents and Case Report

Recent works (Parra et al., 2009, 2010, 2015) have evidenced the critical role of visual short-term memory (STM) binding deficits as a clinical and preclinical marker of Alzheimer’s disease (AD). These studies (and other related reports, see below) show that, in AD, working memory is selectively impaired for tasks requiring binding of multiple elements, but preserved for processing isolated features. Moreover, they suggest a potential role of parieto-temporo-posterior regions in both the neurocognitive deficits of AD patients and STM binding at large. Here we show that direct electrical stimulation of the parietal cortex through invasive electrodes selectively enhances working memory binding, with no effects on feature-level working memory processes.

Memory binding is the function that allows integrating multiple elements of complex events into unified wholes (von der Malsburg, 1999; Baddeley, 2000; Tulving, 2002; Zimmer et al., 2006). STM or working memory binding underpins the temporary retention of arrays of features (e.g., shapes with colors) as integrated complex objects (Treisman and Zhang, 2006). Parra et al. (2010) visual working memory task discriminates between patients with familial AD and preclinical carriers of the causative E280A mutation in the presenilin-1 gene (Lemere et al., 1996) from non-carriers of such a mutation. Notably, despite the impairment in STM binding, asymptomatic carriers and healthy controls did not differ in tasks assessing general memory, attention and executive functions. Impairments in this function are associated with lower white matter integrity in familial Alzheimer disease (Parra et al., 2015). Furthermore, this STM binding deficit is absent in non-AD dementias, such as frontotemporal, vascular, or Lewy body dementias (Della Sala et al., 2012). STM binding seems to place minimal demands on executive functions and appears to be subserved by components of the memory network impaired in AD, but not in other dementias (Della Sala et al., 2012). On the other hand, working memory deficits have been reported in epilepsy patients. For example, temporal lobe epilepsy typically has disabling effects on verbal memory functions. Furthermore, juvenile myoclonic epilepsy and benign epilepsy in children are associated with impaired performance on visual working memory tasks (Elger et al., 2004).

STM binding yields specific activation increases across neural generators that collectively support temporary visual memory for isolated and integrated features. Within the left hemisphere, binding of object features mainly engages the superior and inferior parietal cortex, the fusiform gyrus and the dorsal premotor cortex (Parra et al., 2014). Moreover, research on the temporal dynamics of STM-relevant networks suggest an early role of posterior regions in binding processes (Smith et al., 2017).

Following this evidence, we inferred that stimulation of the posterior parietal cortex (PPC) might offer new opportunities to approach working memory deficits in AD, especially at early stages. However, no study has yet demonstrated a causal role of such a region in STM binding. A unique approach to define necessary hubs in brain networks and infer reliable mechanisms in cognitive neuroscience consists in applying direct intracranial electrical stimulation to single subjects (epilepsy patients implanted with depth electrodes) in specific brain regions to causally modulated cognitive task performance. Here, as part of an ongoing program of research (Chennu et al., 2013; Canales-Johnson et al., 2015; Hesse et al., 2016), we profited from the unique opportunity to produce such causal evidence via single-subject direct intracranial electrical stimulation (Parvizi et al., 2012; Mégevand et al., 2014). Specifically, we stimulated the left parietal cortex (precuneus) during the visual STM task in a patient implanted with intracranial electrodes, and compared the results with a non-stimulated condition and to the performance of a matched control group (for details about the participant and the controls, see “Participants” Section; intracranial recording specifications are offered in “Subject’s Intracranial Recording” Section). The patient presented a specific pattern of working memory deficits (with impairments in the binding condition but not in the shape condition) resembling the typical performance of AD patients on this task (Parra et al., 2009, 2010). This allowed us to test the selective and specific effect observed in the binding condition alone, as a relevant model mirroring the pattern which characterizes AD.

**Figure 1:**
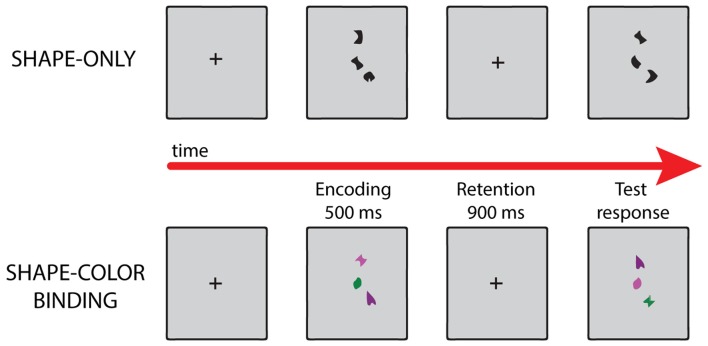
Examples of different trials in each condition of the visual working memory task. For details, see “Experimental Design and Stimuli” Section.

We used the visual working memory task developed by Parra and colleagues (Parra et al., 2010, 2015; Pietto et al., 2016). This task involves two sets of stimuli, one with black shapes, used to evaluate memory for shapes; and a second one with colored shapes, assessing shape-color binding in working memory (see Figure 1). The subject performed the task with and without stimulation. During task performance we directly stimulated the left precuneus with 1 mA at 50 Hz in 2-s intervals (stimulation block). In the control condition (sham block) the patient continued the task under a simulated stimulation (for details see “Experimental Design and Stimuli” and “Stimulation Protocol” Sections, respectively). The subject was not able to distinguish between stimulation and sham blocks.

**Figure 2:**
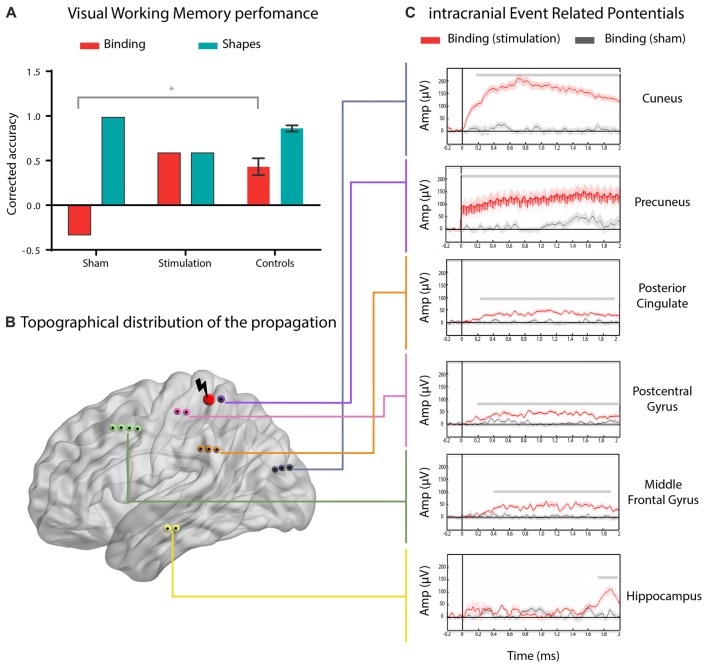
Enhanced working memory binding by direct electrical stimulation of the parietal cortex. **(A)** Mean performance of the subject during the real (posterior parietal cortex, PPC) or sham (sham) stimulation in the shape-only (shapes) and shape-color binding (binding) conditions, compared to the performance of seven matched control participants. Error bars represent standard deviations (SD) from the mean. **(B)** Schematic brain localization of the reported contact sites. Red nodes represent the stimulated site and black nodes show the ROIs were stimulation propagation was detected. **(C)** Intracranial event related potentials (iERP) activity from significant ROIs comparing real and SHAM stimulation in the shape-color binding condition. Shadowed bars around potentials indicate SEM. For visualization purposes, the signal was renormalized, filtered and smoothed. *p* < 0.005, permutation test with bootstrapping and false discovery rate correction, minimum length of windows with significant differences: 100 ms.

Direct stimulation of the parietal cortex induced a selective improvement in STM binding. Relative to controls (“Behavioral Analysis” Section), the subject only reached normal STM binding performance upon stimulation (Crawford’s *t* = 0.20, *p* = 0.84, *Z*_ccc_ = 0.21; Figure 2A). In addition, compared with the SHAM condition, electrical stimulation during the binding condition significantly enhanced performance (Crawford’s *t* = −2.93, *p* = 0.02, *Z*_ccc_ = −3.31; Figure 2A). Moreover, the subject’s performance in the shapes condition did not significantly differ from that of controls, in any of the treatments (PPC: Crawford’s *t* = 1.33, *p* = 0.23, *Z*_ccc_ = −1.42; SHAM: Crawford’s *t* = 2, *p* = 0.08, *Z*_ccc_ = 2; Figure 2A). To assess the impact and spread of the electrical stimulation, we compared intracranial event-related activity in all the recording electrodes between stimulated and SHAM trials (Figure 2B; for data pre-processing and processing see “Signal Preprocessing and Data Quality” Section, respectively). Propagation of the excitation was widely distributed across the left hemisphere. The intracranial event related potentials (iERP) showed maximal effects in parietal regions, followed by the cuneus, the posterior cingulate, the postcentral gyrus, the middle frontal gyrus, and the hippocampus, in a constant decreasing fashion (all *p*s < 0.005, permutation test with bootstrapping and false discovery rate correction; Figure 2C).

Meta-analytic evidence suggests an early compromise of parietal networks in AD (Jacobs et al., 2013). Here, using a causal stimulation-based method, we offer the first evidence of a selective and causal involvement of the parietal cortex in working memory binding. Such selectivity is reinforced by the null effect yielded by the same stimulation on STM processing of individual features (shapes only, control of task). Although there is a tendency to decrease in shapes accuracy with the precuneus’ stimulation, we are not able to explain this with our present data. However, we could speculate that this diminution might reflect resource distribution effect, such increased allocation of resources to “binding” processes would deplete resources available for the “encoding” process. Furthermore, the improvement of STM binding was triggered by stimulation of the precuneus, which in turn induced a spreading of activation throughout a frontoparietal and hippocampal network including the posterior network previously related to visual memory binding (Parra et al., 2014). These regions are also part of the top-down attentional control network (Gazzaley and Nobre, 2012). Importantly, this binding-specific improvement is not due to a task-learning effect, since real stimulation was performed before the SHAM condition (no-stimulation control). Together with previous findings, these results provide preliminary but promising findings for a new agenda aiming at evaluating behavioral changes upon parietal stimulation in AD through TMS or tDCS. The next step will be to perform systematic stimulation studies targeting various specific and unspecific posterior hubs to assess how critical the parietal cortex is for STM binding. In this sense, we hypothesize that the stimulation of the left PPC would specifically improve performance on this task. Future study designs should take into account the method, type, duration and number of sessions of the stimulation protocol to modulate STM binding processes. In particular, future non-invasive stimulation protocols should be extended in their length and their number of trials. Furthermore, it would be of great interest to assess physiological changes related to the stimulation protocol, so as to better identify the mechanisms underlying STM binding. Furthermore, tDCS is a safe, non-invasive method that could be used to improve memory impairments or diminish seizure frequency in in drug-resistant epilepsy. In patients with intractable lateral frontal lobe epilepsy, Karvigh et al. (2017) found that cathodal HD-tDCS of the epileptogenic zone significantly improved attention and working memory immediately and 1 month after stimulation. Moreover, Tekturk et al. (2016) applied modulated cathodal stimulation (2 mA for 30 min on three consecutive days) to patients diagnosed with mesial temporal lobe epilepsy with hippocampal sclerosis, and found that more than at least 8 out of 10 patients had more than 50% decrease in seizure frequency. Therefore, non-invasive brain stimulation might be a promising tool to attenuate or delay memory deficits in AD and may be used as an additional treatment option for refractory epilepsies. However, further studies are necessary to assess these approaches.

Intracranial recordings are exceptional in humans and provide a unique opportunity to obtain causal stimulation-based evidence with high spatiotemporal resolution, they have, however, important limitations. While we have accounted for the known caveats of intracranial EEG recordings by adopting several measures (see “Signal Preprocessing and Data Quality” Section), future studies could further test our conclusions while circumventing method-specific limitations.

As reported in the pioneering work (Penfield and Boldrey, 1937), electrical stimulation has been used both for clinical purposes and to causally probe brain mechanisms. The evidence for electrical currents to spread through white matter along functionally well-defined brain networks (Duffau et al., 2005; Tolias et al., 2005) supports our result that a wide network underlies specific visual working memory mechanisms. Direct cortical stimulation becomes much more than a blunt tool to modulate a simple area, and turns into an unique causal instrument to probe into the process of the investigated function.

Finally, given the usefulness of the STM binding task as a marker of prodromal AD, and alongside the potential to improve STM binding by stimulation, our results open a new area of research centered in the non-invasive brain stimulation of the PPC in clinical and preclinical populations. Future studies with this approach may shed light on functional restoration options for AD patients and subjects at risk for the disease (mild cognitive impairment, MCI), paving the way for new treatments to delay the development of neurocognitive deficits associated with this pathology.

## Materials and Methods

### Data Acquisition and Protocol Design

#### Participants

As part of an ongoing protocol (Chennu et al., 2013; Canales-Johnson et al., 2015; Hesse et al., 2016), we recruited one patient with intractable epilepsy who was offered surgical intervention to alleviate his condition. The subject was an 18-year-old, right-handed male who had completed high school and suffered from drug-resistant epilepsy since the age of six. He was attentive and cooperative throughout the task. In addition, we recruited a control group comprised of seven healthy male participants matched with the patient for age (*M* = 18.57, *SD* = 1.27; *t* = −0.35, *p* = 0.70) and years of education (*M* = 12.71, *SD* = 1.25; *t* = −0.53, *p* = 0.61). None of these subjects reported a history of psychiatric or neurological disease. This study was carried out in accordance with the recommendations of the Declaration of Helsinki, as well as the Guidelines of the Ethics Committee of INECO (approved protocol number FONCyT-PICT 2012-0412 and 2012-1309). All subjects gave written informed consent in accordance with the Declaration of Helsinki.

#### Subject’s Intracranial Recording

Direct cortical recordings were obtained from semi-rigid, multi-lead electrodes implanted in the patient’s brain. The electrodes were 0.8 mm in diameter and consisted of 5, 10 or 15 2-mm wide contact leads placed 1.5 mm apart from each other (DIXI Medical Instruments). We used a Micromed video-SEEG monitoring system which records as many as 128 depth-EEG electrode sites simultaneously. Recordings were obtained from 127 sites and sampled at 512 Hz. The data were collected from the precuneus, the cuneus, the hippocampus, the posterior cingulate and the postcentral gyrus. The recordings obtained were distal to the epileptogenic foci, and no single recording site presented epileptogenic activity (see below). We also obtained post-implantation MRI and CT scans were obtained from each patient. Both volumetric images were affine registered and normalized on the SPM8 MATLAB toolbox. Using MRIcron, we established the coordinates of each contact site and their respective Brodmann areas.

#### Experimental Design and Stimuli

The task assessed memory for shapes and combinations of shapes and colors. Stimuli were randomly selected from a set of eight shapes and eight colors and presented as individual features or as features combined into integrated objects. Each type of stimulus was presented in a separate condition. Two experimental conditions were used, each consisting of 32 test trials, leading to a total of 64 test trials. Trials were fully randomized and, for the control participants, conditions were delivered in a counter-balanced order. In the “Shapes” condition, arrays of shapes were presented in the study display. In the test display for the “different” trials, two new shapes from the study array were replaced with two new shapes (Figure 1). Hence, in these conditions, participants were required to detect changes in individual features. In the “Shape-color binding” condition, the study display showed combinations of shapes and colors. In the test display for “different” trials, two shapes swapped the colors in which they had been shown in the study display (Figure 1). Hence, detection of this change relied on shape-color bindings. No shape or color was repeated within a given array. Fifty percent of the test trials were “same” trials (the study and test displays presented identical items) and 50% were “different” trials.

#### Stimulation Protocol

Electrical stimulation was delivered in bipolar square waves between two adjacent electrode contacts in the left precuneus, 54 (−4 −60 56, MNI coordinates) 55 (−2 −60 56, MNI coordinates). Stimulation occurred at 1 mA for the real stimulation condition and at 0 mA for the control SHAM condition using a 200 ms pulse width at a frequency of 50 Hz, during 2 s. Each condition involved 10 trials under real stimulation and another 10 under SHAM stimulation. The stimulation began at the onset of the study display and continued through the test display. First we performed the real and SHAM stimulation (in that order) for the shape-color binding condition and then for the shape-only condition. EEG signals were simultaneously monitored before and after discharges. Electrodes and trials compromised by seizures or leading to epileptic activity were excluded. The subject was asked to describe any perceptual or physical changes he experienced during or after each stimulation trial.

### Data Analysis

#### Behavioral Analysis

As in previous studies (Parra et al., 2010, 2015; Pietto et al., 2016), recognition during the visual working memory task was calculated by subtracting the proportion of false alarms from the hits. The subject’s indexes were compared to those of a control sample through a modified two-tailed *t* test (Crawford and Garthwaite, 2002; Crawford et al., 2009, 2011). This methodology allows the assessment of significance by comparing test scores of one or several individuals with norms derived from small samples. This modified test is robust for non-normal distributions, presents low values of type I error, and has already been reported in single case studies (Straube et al., 2010). The alpha level was set at *p* < 0.05.

#### Signal Preprocessing and Data Quality

Several measures were adopted to circumvent the caveats of data obtained from an epileptic patient. We excluded channels in epileptogenic foci, used stringent inclusion criteria for the remaining channels, and ensured the absence of neuroanatomical abnormalities or major cognitive deficits in the subject. We discarded the contact sites that presented pathological waveforms. Electrodes with epileptic activity were discarded upon visual identification by two professional neurologists (MCG and JA). Moreover, we discarded channels whose values exceeded five times the signal’s mean and/or consecutive signal samples exceeding five standard deviations (SD) from the gradient’s mean (Chennu et al., 2013; Hesse et al., 2016). A total of 83 contact sites remained after applying these criteria, and all of them were processed. Then, the data were referenced to the mean value of the non-stimulated sites (averages of such sites were subtracted from each recording). Finally, the data were segmented into 2000 ms epochs, including a −200 to 0 ms pre-stimulus baseline period. The epochs were baseline corrected.

## Author Contributions

AB and AI designed the study. AB, EH, LS, EPM, MdCG, JA, FA, AL, TAB, MZ, MP, AMG and AI carried out and analyzed the experiments. AB and AI wrote the article. AI and AB conceived the study and wrote the final article, together with the other authors. All authors have approved the manuscript.

## Conflict of Interest Statement

The authors declare that the research was conducted in the absence of any commercial or financial relationships that could be construed as a potential conflict of interest.
